# Arabidopsis AGO4 loses its Cajal body localization when heterologously expressed in *Nicotiana benthamiana*

**DOI:** 10.1080/19420889.2022.2051843

**Published:** 2022-03-24

**Authors:** Liping Wang, Rosa Lozano-Duran

**Affiliations:** a Shanghai Center for Plant Stress Biology, Center for Excellence in Molecular Plant Science, Chinese Academy of Sciences, Shanghai, China; b Department of Plant Biochemistry, Centre for Plant Molecular Biology (ZMBP), Eberhard Karls University, Tübingen, Germany

**Keywords:** AGO4, Cajal body, *Nicotiana benthamiana*, *Arabidopsis thaliana*

## Abstract

In plants, the RNA-directed DNA methylation (RdDM) pathway plays a major role in establishing DNA methylation. At least some components of the RdDM machinery, including the central component AGO4, are known to concentrate in a subnuclear compartment called the Cajal body in the model plant *Arabidopsis thaliana*. The molecular underpinnings of Cajal body localization, however, have remained elusive so far. Here, we found that Arabidopsis AGO4 (AtAGO4) fused to GFP does not present its typical Cajal body localization, when transiently expressed in *Nicotiana benthamiana*. Nevertheless, the endogenous AGO4 protein from *N. benthamiana* shows a clear accumulation in the Cajal body. Thus, our results suggest that the Cajal body localization of AtAGO4 requires specific molecular machinery that cannot be replaced by orthologues in *N. benthamiana*. This study presents an experimental system that could lead to mechanistic insights into the targeting of proteins to and localization in the Cajal body in plants.

## Introduction

DNA methylation in cytosine residues is a conserved epigenetic mark essential for genome stability, defense, and development in plants. DNA methylation in all sequence contexts is established *de novo* by the RNA-directed DNA methylation (RdDM) pathway [1]. In the canonical RdDM pathway, a single-stranded non-coding RNA (ncRNA) transcribed by DNA-directed RNA polymerase IV (Pol IV) is converted into a double-stranded RNA (dsRNA), which is processed into 24-nucleotide (nt) siRNAs by DICER-LIKE 3 (DCL3). 24 nt siRNAs are loaded into ARGONAUTE 4 (AGO4), and the AGO4-siRNA complexes are directed to the RdDM-targeted loci by base-pairing with the ncRNA transcribed by Pol V. Then, the resulting complex recruits the DNA methyltransferase DRM2 to catalyze DNA methylation of adjacent DNA sequences [^2–6^].

Cajal bodies are ubiquitous subnuclear organelles in both plant and animal cells, and are implicated in maturation of ribonucleoprotein complexes [7]. In Arabidopsis, several RdDM core components, such as AGO4 and NRPE1 (the biggest subunit of Pol V), have been shown to concentrate in the Cajal body, suggesting that the Cajal bodies are potential sites for assembly of protein complex in RdDM pathway [^8–10^]. Our previous work shows that AGO4 proteins from the experimental *Solacaneae Nicotiana benthamiana* also localize in the Cajal body, and that methylation of the tomato yellow leaf curl virus (TYLCV) genome occurs in a Cajal body-dependent manner [11]. These findings suggest that the subnuclear localization of AGO4 is evolutionarily conserved, and relevant for its function in the RdDM pathway. However, the molecular underpinnings of the AGO4 localization in the Cajal body are unknown so far. Does AGO4 require specific protein-protein interactions for its localization in the Cajal body? If so, are these interactions conserved in different plant species? Is there a specific motif determining Cajal body protein localization?

## Results and discussion

**Figure 1.**
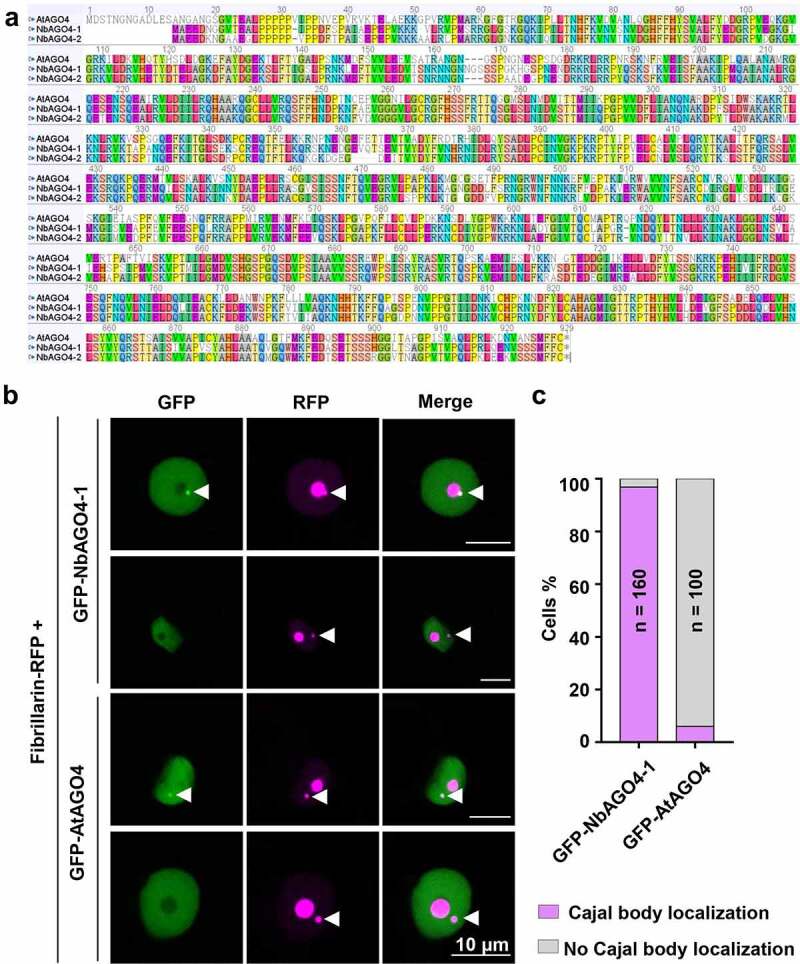
AtAGO4 does not localize in the Cajal body when transiently expressed in *N. benthamiana*. A. Alignment of AGO4 proteins from Arabidopsis and *N. benthamiana*. The alignment was performed by using ClustalW. The identities between AtAGO4 and NbAGO4-1, AtAGO4 and NbAGO4-2, and NbAGO4-1 and NbAGO4-2 are 73.5%, 72.5%, and 86.8%, respectively. B. Subcellular localization of GFP-AtAGO4 and GFP-NbAGO4-1 in *N. benthamiana* leaves. Fibrillarin-RFP is used as a nucleolus and Cajal body marker. GFP-AtAGO4 or GFP-NbAGO4-1 were co-expressed with Fibrillarin-RFP in *N. benthamiana* leaf epidermal cells by *A. tumefaciens*-mediated transient gene expression. Confocal images were taken two days after infiltration. Arrowheads indicate the position of the Cajal body. Bar, 10 μm. This experiment was repeated three times with similar results. C. Quantification of Cajal body localization of GFP-NbAGO4-1 or GFP-AtAGO4 in *N. benthamiana* leaves.

With the aim of gaining insight into the molecular mechanisms determining the Cajal body localization of proteins, more specifically of AGO4, in plants, we sought out to determine the subcellular localization of AGO4 from Arabidopsis (AtAGO4) and *N. benthamiana* (NbAGO4-1 and NbAGO4-2) in transiently transformed cells of the latter. First, we compared the sequence of these orthologous proteins. As shown in Figure 1a, the protein sequences are highly similar among AGO4 orthologs from these two species, and the identities are 73.5% (AtAGO4 vs NbAGO4-1), and 72.5% (AtAGO4 vs NbAGO4-2), respectively (Figure 1a); interestingly, AtAGO4 has a 13-aa N-terminal extension not present in the *N. benthamiana* proteins. We next constructed a vector to express GFP-fused AtAGO4 under the control of a 35S promoter. The GFP-fused AGO4 from *N. benthamiana* (GFP-NbAGO4-1) was used as positive control, since the Cajal body localization of NbAGO4-1 has been previously described [11]. Then we transiently co-expressed GFP-AtAGO4 or GFP-NbAGO4-1 with the nucleolus and Cajal body marker Fibrillarin-RFP [12,13] in *N. benthamiana* epidermal cells by *Agrobacterium tumefaciens*-mediated transformation. As shown in Figure 1b, both GFP-AtAGO4 and GFP-NbAGO4 are distributed throughout the nucleoplasm and absent from the nucleolus. Remarkably, in contrast to GFP-NbAGO4, which co-localizes with Fibrillarin-RFP in the Cajal body in most cases, GFP-AtAGO4 does not localize in the Cajal body in 94% cells in this experimental system (Figure 1b, 1c).

This result suggests that the molecular mechanisms directing AtAGO4 to the Cajal body in Arabidopsis are not functional in *N. benthamiana*. We envision two alternative scenarios to explain this observation: (1) AtAGO4 contains a putative signal to guide it to the Cajal body, but this signal is not recognized in *N. benthamiana*. Based on the alignment in Figure 1a, the N-terminal part of AGO4 is the most highly divergent part of the protein; therefore, we speculate that this putative signal might be located at the N-terminus of AtAGO4; (2) AtAGO4 reaches the Cajal body by interacting with Cajal body-resident proteins, such as Coilin, Fibrillarin, or U2B [9,12,14], or “enabling” proteins that facilitate this localization, but such interaction(s) is/are not maintained in *N. benthamiana*.

The transient expression system in *N. benthamiana* is widely used to study the localization and interactions of proteins of interest in a simple, rapid, and efficient manner. However, these results serve as a cautionary note: it is important to keep in mind that the differences between species may affect the behavior of the proteins under study, giving rise to artifactual subcellular localizations.

The AtAGO4/NbAGO4/*N. benthamiana* system offers a framework to investigate the molecular mechanisms underlying the Cajal body targeting and localization of AGO4. The comparative analysis of the AGO4 protein sequences, as well as of the differential interactome of AtAGO4 and NbAGO4-1 in *N. benthamiana* and Arabidopsis, may shed light onto the elusive cellular strategies for Cajal body protein targeting.

## Material and methods

### Cloning and plasmids

The full-length coding sequence of AtAGO4 (gene ID, AT2G27040.1) was amplified by PCR (Fw: CACCATGGATTCAACAAATGGTAACG, Rv: TTAACAGAAGAACATGGAGTTGGCG) using cDNA as a template, and cloned into pENTR-D/TOPO (Invitrogen) following the manufacturer’s instructions. pENTR-D/TOPO-NbAGO4-1 has been previously described [11]. The binary plasmids to express N-terminal GFP fused AtAGO4 or NbAGO4-1 were generated by Gateway-cloning the AtAGO4 or NbAGO4-1 coding sequence from pENTR-D/TOPO entry vector into pGWB506 [15]. The clones to express Fibrillarin-RFP have been previously described [11,13].

### Plant materials and growth conditions

*N. benthamiana* plants were grown in a controlled growth chamber under long day conditions (LD, 16 hr light/8 hr dark) at 25°C.

### A. tumefaciens-mediated transient gene expression in N. benthamiana

All binary plasmids were transformed into *A. tumefaciens* (strain GV3101), which were liquid-cultured in LB with appropriate antibiotics at 28°C overnight. Bacterial cultures were then centrifuged at 4000 g for 10 min and resuspended in the infiltration buffer (10 mM MgCl_2_, 10 mM MES pH 5.6, 150 mM acetosyringone) and adjusted to an OD_600_ = 0.25. For co-expression experiments, the different *A. tumefaciens* suspensions were mixed at 1:1 ratio and then incubated at room temperature for 2–4 hours in the dark before they were infiltrated to 4-week-old *N. benthamiana* plant leaves.

## Confocal imaging assays

Confocal imaging for co-localization of Fibrillarin-RFP and GFP-AtAGO4 or GFP-NbAGO4-1 upon transient expression in *N. benthamiana* epidermal cells was performed on a Leica TCS SP8 point scanning confocal microscope using the pre-set sequential scan settings for GFP (Ex: 488 nm, Em: 500–550 nm), and RFP (Ex: 561 nm, Em: 600–650 nm).
